# Supplementary material for: Amplified warming of North American cold extremes linked to human-induced changes in temperature variability
Source: Nat Commun. 2024 Jul 12;15:5864. doi: 10.1038/s41467-024-49734-8 (PMC11245492; doi:10.1038/s41467-024-49734-8)
Supplement: Supplementary file 1 — Supplementary Information [file 41467_2024_49734_MOESM1_ESM.pdf]

# Supplementary Information for: “Amplified warming of North American cold extremes linked to human-induced changes in temperature variability”

Russell Blackport* and John C. Fyfe

Canadian Centre for Climate Modelling and Analysis, Environment and Climate Change Canada, Victoria, BC, Canada

*Corresponding author email: russell.blackport@ec.gc.ca

## Supplementary Figures

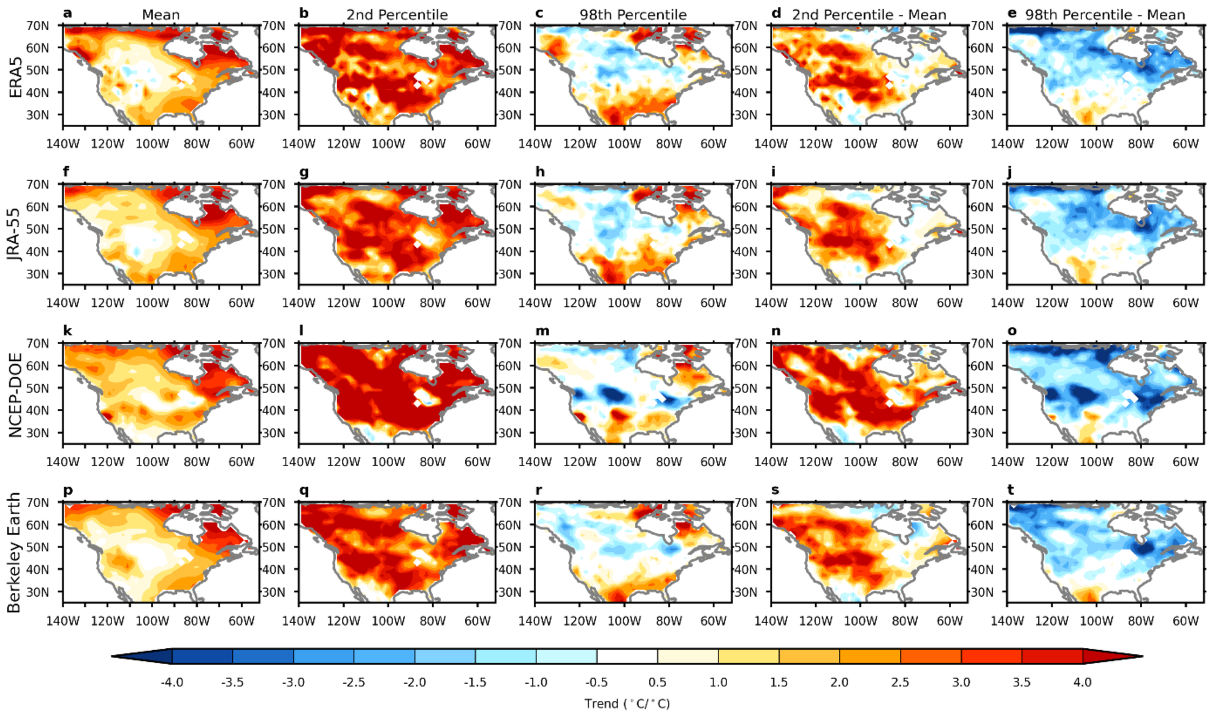


**Supplementary Figure 1: Trends in mean and extreme temperatures in different reanalysis and observation datasets.** The 1980-2022 winter temperature trends in ERA5 reanalysis for the mean (**a**) , 2$^{nd}$ percentile (**b**), 98$^{th}$ percentile (**c**) , the difference between the 2$^{nd}$ percentile and the mean (**d**), and the difference between 98$^{th}$ percentile and the mean (**e**). **f-j** As in **a-e**, but for JRA-55 reanalysis. **k-o** as in **a-e**, but for NCEP-DOE reanalysis 2. **p-t** As in **a-e**, but for the Berkeley Earth gridded daily observations. Note that **a-e** is repeated from Fig 1a-e. All trends are divided by the corresponding global, annual, mean temperature trend, so that the units of the trends are °C per °C of global warming.

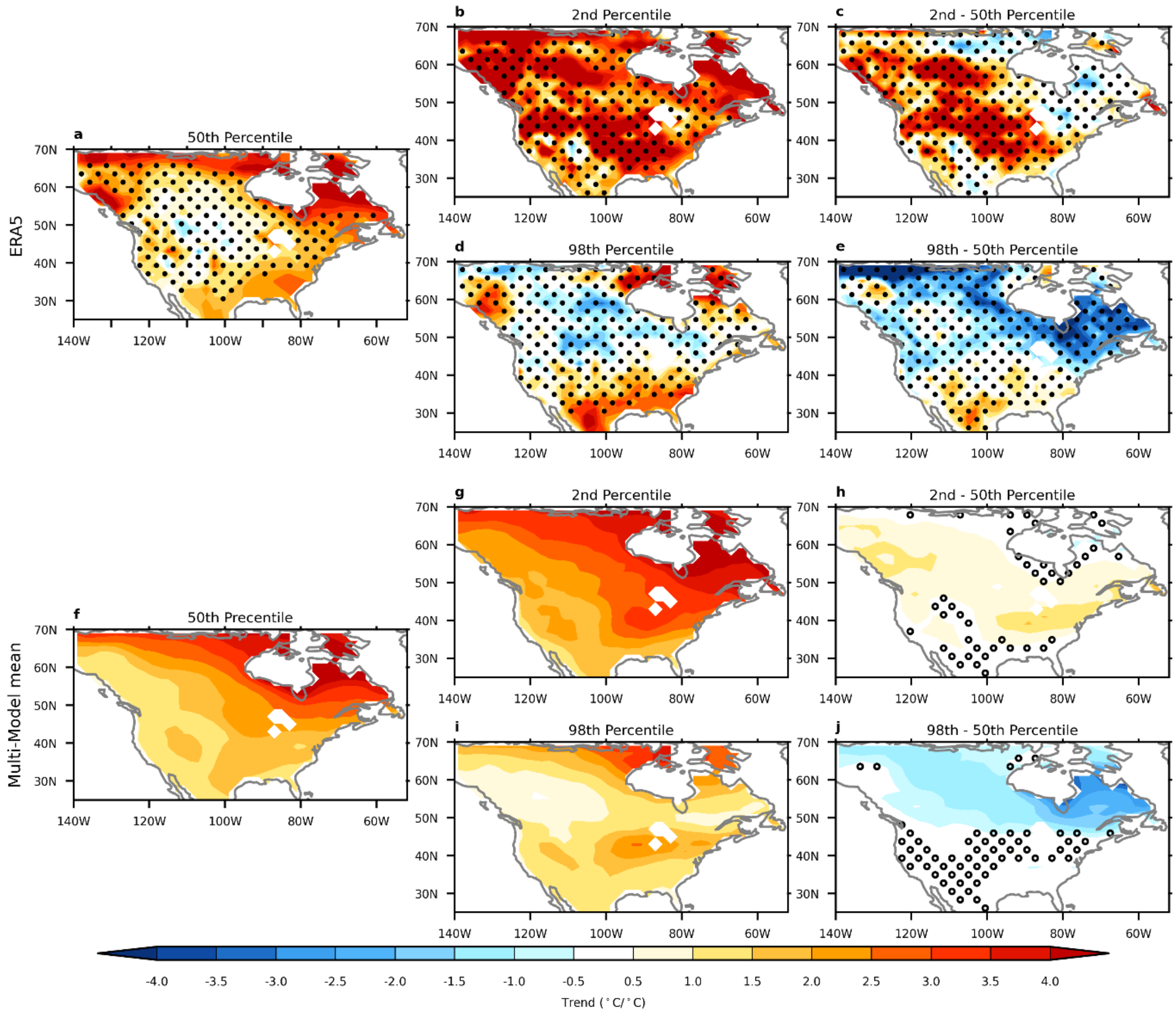


**Supplementary Figure 2: Trends in winter 50$^{th}$ percentile and extreme temperatures. a-e** The 1980-2022 winter near-surface temperature trends in ERA5 reanalysis for the 50$^{th}$ percentile (**a**), 2$^{nd}$ percentile (**b**), the difference between the 2$^{nd}$ percentile and the 50$^{th}$ percentile (**c**), 98$^{th}$ percentile (**d**), and the difference between the 98$^{th}$ percentile and the 50$^{th}$ percentile (**e**). **f-j** As in **a-e**, but for the multi-model mean. All trends are divided by the corresponding global, annual, mean temperature trend, so that the units of the trends are °C per °C of global warming. The stippling in **a-e** represents where the ERA5 trends are not statistically significant using a bootstrap approach after controlling for the false discovery rate of 0.1. The stippling in **f-j**

indicates where more than one of the models disagree on the sign of the trend in the ensemble mean.

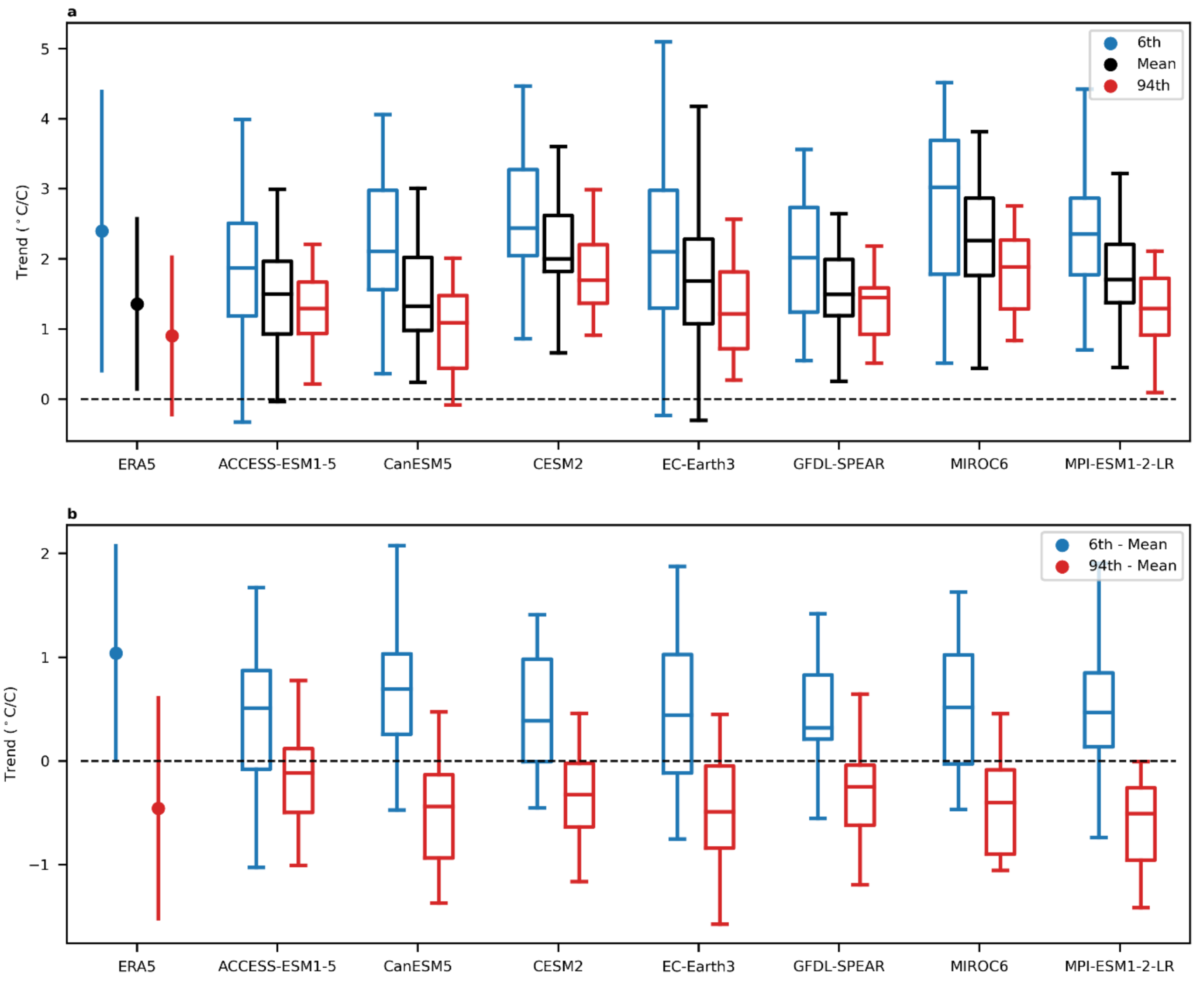


**Supplementary Figure 3: Comparison of trends in reanalysis to the model ensemble spread for less extreme trends. a** The magnitude of the 1980-2022 trend in winter 6$^{th}$ percentile temperature (blue), winter mean temperature (black) and 94$^{th}$ percentile temperature (red) from ERA5 (dots). Trends are averaged between 140°-52°W and 30-52°N, land only. The error bars for ERA5 represent the 2.5-97.5% range is calculated using a bootstrapping approach. The box and whisker plots represent the ensemble spread in trends from each of the seven models. The

box represents the inner quartile range, the whiskers represent the 2.5 - 97.5% range of trends, and the line represents the median trend. All trends are divided by the corresponding global, annual, mean temperature trend, so that the units of the trends are °C per °C of global warming. **b** As in **a**, but for the difference between the 6th percentile and mean trends (blue) and the difference between the 94th percentile and the mean trend (red).

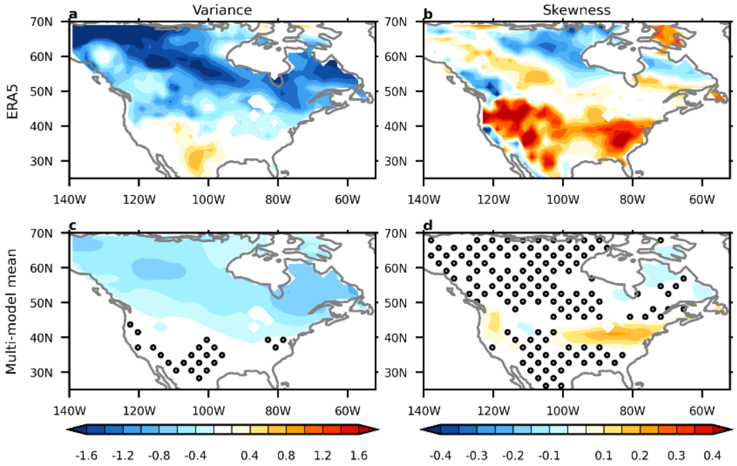


**Supplementary Figure 4: Trends in variance and skewness. a** The 1980-2022 trends in ERA5 winter temperature variance (**a**), and skewness (**b**). **c**, **d** As in **a**, **b** but for the multi-model mean. All trends are divided by the corresponding global, annual, mean temperature trend, so that the units of the variance trends are °C$^2$ per °C of global warming. The stippling indicates where more than one of the models disagree on the sign of the trend in the ensemble mean.

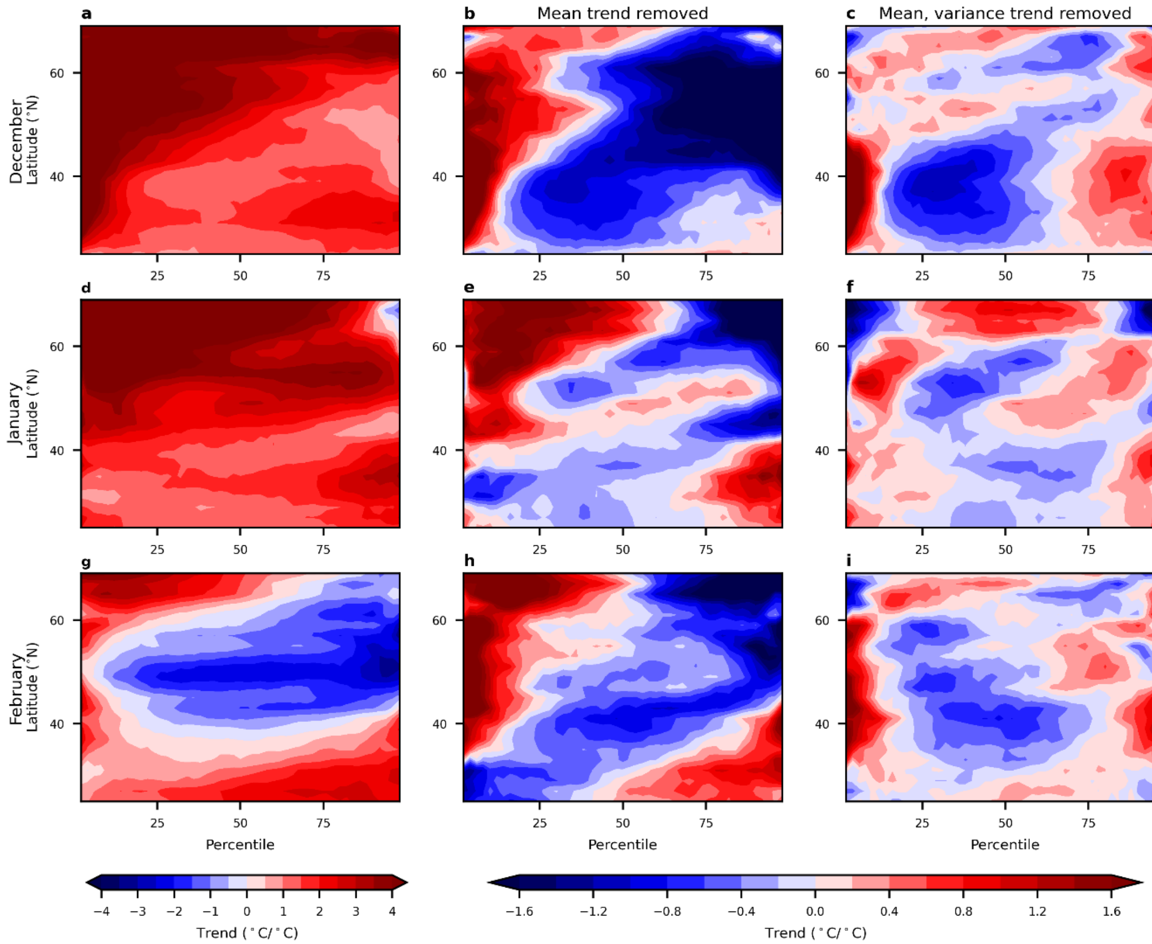


**Supplementary Figure 5: Zonal mean of winter temperature trends as a function of percentile for individual months. a** Zonal mean (averaged between 140° and 52°W, land only) of December temperature trends as a function of percentile and latitude for ERA5 over 1980-2022. **b** as in **a**, but with the winter mean trend subtracted from the trend. **c** As in **b**, but with both trend in mean and variance trend subtracted from trends. **d-f** As in a-c, but for January. **g-i** As in **a-c**, but for February. All trends are divided by the corresponding global, annual, mean temperature trend, so that the units of the trends are °C per °C of global warming.

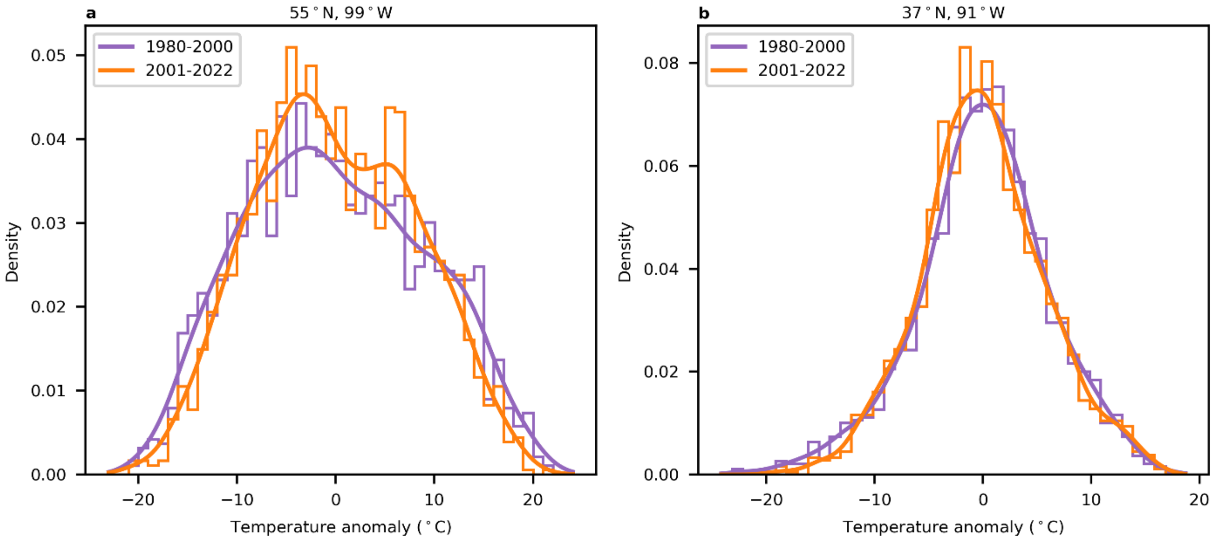


**Supplementary Figure 6: ERA5 temperature distributions at representative grid points.** Histograms of the winter temperature anomalies during the 1980-2000 (purple) and 2001-2022 periods (orange) for grid points 55°N,99°W (**a**) and 37°N, 91°W(**b**). These grid points are representative of the regions where the amplified warming of extreme cold is primarily linked to change in variance (**a**) and where the amplified warming is primarily linked to changes in higher moments of the distribution (**b**). The histograms are smooth by estimating the probability density function. The mean temperature for each time period is removed, so the difference between the time periods reflect only the changes shape of the distributions and not a shift in mean.

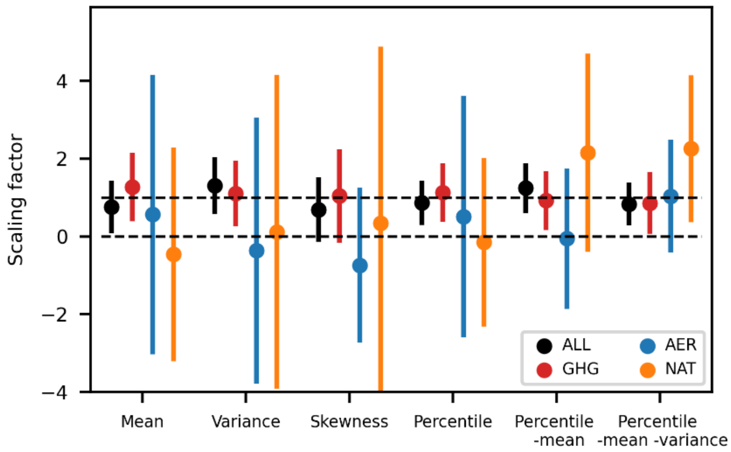


**Supplementary Figure 7: Scaling factors from the three-signal detection and attribution analysis.** The scaling factors for the multi-model mean fingerprint of the trend in mean, variance, skewness, trends as a function of percentile, trends as a function of percentile with the mean trend removes, and trends as a function of percentile with both the mean and variance removed. Scaling factors are plotted for the all historical forcing (black) and the three GHG, AER and NAT. For the all historical forcing, the scaling factors are calculating using the multi-model mean fingerprint from only the three models that have the single forcing large ensembles available. The uncertainty on the scaling factors represents the 5-95th percentile range calculated from internal variability.
